# Characterising the longer-term impacts of highly pathogenic avian influenza outbreaks prior to 2020 on wild bird populations

**DOI:** 10.1371/journal.pone.0360235

**Published:** 2026-11-11

**Authors:** Susannah Gold, Fraser Bell, Emma Sheard, Abbas Ashoori, Luka Božič, Sebastian Bugariu, Achilles Byaruhanga, Svilen Cheshmedzhiev, Wolfgang Fiedler, Anja Globig, El-Yamine Guergueb, Yuko Haraguchi, Bernd Heinze, Young-Jun Kim, Erik Kleyheeg, Bernd Koop, Oliver Krone, Azwianewi Makhado, Pascal Orabi, Loïc Palumbo, Simon Piro, Gwenaël Quaintenne, Laura Roberts, Jean-Paul Roux, Roy Slaterus, Anne van de Wiele, Will Peach

**Affiliations:** 1 RSPB Centre for Conservation Science, RSPB, The Lodge, Sandy, United Kingdom; 2 Centre for Ecology and Conservation, University of Exeter, Penryn, Cornwall, United Kingdom; 3 Department of the Environment, Gilan Provincial Office, Rasht, Iran; 4 DOPPS – BirdLife Slovenia, Ljubljana, Slovenia; 5 Societatea Ornitologica Romana/Romanian Ornithological Society, Bucharest, Romania; 6 Nature Uganda, Kampala, Uganda; 7 National Museum of Natural History, Bulgarian Academy of Sciences, Sofia, Bulgaria; 8 Bulgarian Society for the Protection of Birds, Sofia, Bulgaria; 9 Max Planck Institute of Animal Behavior, Radolfzell, Germany; 10 Friedrich Loeffler Institute, Greifswald, Germany; 11 Laboratoire Valorisation et Conservation des Ecosystèmes Arides (LVCEA), Faculté des Sciences de la Nature et de la Vie et Sciences de la terre, Université de Ghardaïa, Ghardaïa, Algeria; 12 Izumi City Crane Museum, Izumi Crane Park, Kagoshima, Japan; 13 Landesamt für Umwelt, Naturschutz und Geologie Mecklenburg-Vorpommern (LUNG), Güstrow, Germany; 14 Division of Restoration Research, Research Center for Endangered Species, National Institute of Ecology, Yeongyang-gun, Gyeongsangbuk-do, Republic of Korea; 15 Sovon Dutch Centre for Field Ornithology, Nijmegen, The Netherlands; 16 Ornithologische Arbeitsgemeinschaft für Schleswig-Holstein and Hamburg, Winnert, Germany; 17 Department of Wildlife Diseases, Leibniz Institute for Zoo and Wildlife Research, Berlin, Germany; 18 Branch Oceans & Coasts, Department of Forestry, Fisheries and the Environment, Cape Town, South Africa; 19 FitzPatrick Institute of African Ornithology, University of Cape Town, Cape Town, South Africa; 20 Center for Polar Research, Shanghai Ocean University, Shanghai, China; 21 LPO France, Rochefort, France; 22 SantéAgri, Direction de la Recherche et de l’Appui Scientifique (DRAS), Office Français de la Biodiversité (OFB), Orléans, France; 23 Abteilung Naturschutz und Naturparke, Landesamt für Umwelt, Naturschutz und Geologie M-V, Güstrow, Germany; 24 Department of Agriculture, Western Cape Government, Elsenburg, South Africa; 25 SEACODE Research Group, Lüderitz, Namibia; 26 Direction de la Recherche et de l’Appui Scientifique (DRAS), Office Français de la Biodiversité (OFB), Villeneuve-de-rivière, France; Universidad Cooperativa de Colombia, COLOMBIA

## Abstract

Since 2020, there has been a global escalation of the impacts of highly pathogenic avian influenza (HPAI) on wild bird species. However, the population-level consequences of this disease remain poorly understood, despite repeated outbreaks affecting wild birds over the past two decades. To address this, we collated population counts and mortality data from 40 historical cases where HPAI mass-mortality occurred in wild bird populations prior to 2020. We characterised local impacts of these outbreaks based on i) initial change in population count from the last count pre-outbreak to the first count post-outbreak, ii) longer-term change in average population counts, iii) change in population trend, and iv) time taken to recover to the pre-outbreak baseline. Following outbreaks, initial population count declines were observed in most cases, with a median decrease of 17% (Inter-quartile range −35% to 5%). Yet in the longer-term, significant reductions in average population counts were detected in only eight of the 40 cases, and a significant decrease in population trend in only two cases. Overall, in the majority of local populations, outbreaks resulted in either limited impacts on population counts or short-term reductions followed by recovery. However, in 25% of cases (10/40) initial declines were not recovered within the monitoring period (4–17 years after the initial outbreak). Initial declines were more apparent in gulls and terns; however, impacts were seen in local populations across a range of taxa. Given that the scale of impacts of HPAI has changed dramatically since 2020, our findings support the need for disease surveillance to be combined with effective population monitoring in order to identify species impacted from the ongoing outbreaks. Further research is also needed to identify strategies for minimising impacts of HPAI, and to understand factors limiting recovery in affected populations.

## Introduction

Wildlife disease is increasingly recognised as a mechanism contributing to population declines. In recent decades, declines in wildlife populations have been attributed to diseases such as chytridiomycosis in amphibians [1], white-nose syndrome in North American bats [2] and trichomonosis in finches in the UK [3,4]. The threat of infectious diseases to population persistence is particularly severe when generalist pathogens, capable of infecting a wide range of host species, emerge and spill over into new, immunologically naïve hosts [5]. This is evident for highly pathogenic avian influenza (HPAI) in wild bird populations, a geographically widespread disease capable of infecting a broad range of both avian and mammalian hosts. While avian influenza viruses circulate globally in wild bird populations in low pathogenicity forms, notably within Anseriformes and Charadriiformes [6], the emergence of high-pathogenicity strains can cause systemic disease and mass mortality in affected species [7].

Avian influenza viruses are grouped into sub-types based on their two surface proteins, the haemagglutinin (HA) protein, which facilitates viral attachment and entry into host cells, and the neuraminidase (NA) protein, which enables release of the virus from the cell. Emergence of HPAI occurs when low-pathogenicity viruses of H5 or H7 sub-types mutate into high-pathogenicity forms, through changes to the HA protein that facilitate systemic viral replication. These conversion events have most commonly been reported in commercial poultry [8]. The H5 sub-type viruses currently in circulation are part of the goose/Guangdong H5 lineage, which was first detected in poultry in China in 1996, and has subsequently diversified into multiple different clades. These viral clades are classified based on the phylogeny of the H5 gene, with sub-clades nested within higher level clades (e.g., 2.3.4.4). The virulence of a specific virus within this lineage will vary depending on a number of factors, including the specific HA and NA genotype, and the constellation of internal genes. Different viral genotypes within a clade can also exhibit different levels of adaptation to different host species. Therefore, although general patterns are seen within clades, with the current clade 2.3.4.4b able to infect a wide range of species, often with high mortality [9], the impacts of an outbreak in a particular population will depend on the specific host-virus combination [10].

Since its emergence in 1996, viral strains from the goose/Guangdong lineage, in particular of clade 2, have established sustained transmission in poultry and wild birds. In 2005–2006, a significant resurgence event occurred of clade 2.2 virus, with a mass mortality event in wild birds at Qinghai Lake in China [11], and subsequent spread causing outbreaks in poultry and wild birds across Asia, Europe, and into Africa [12]. Waterfowl were primarily affected, but cases were also reported in birds of prey [13]. A further clade, 2.3.2, emerged in 2009 and circulated in poultry and wild birds in Asia [14]. Subsequent spread (of sub-clade 2.3.2.1), into Eastern Europe in 2010 and 2015 also resulted in mass mortality events in wild birds, including Dalmatian pelicans (*Pelecanus crispus*) in 2015 [15,16]. From 2014, clade 2.3.4.4 emerged, with this clade appearing to have an improved ability to spread in wild birds, leading to a further resurgence event in 2014–2015 [12]. In 2016, clade 2.3.4.4b became the dominant clade in circulation, and was responsible for an epizootic in 2016–2017. In Europe, outbreaks of clade 2.3.4.4b H5N8 in the autumn and winter of 2016–2017 led to mass mortality in wintering waterfowl [17]. Subsequent spread into Africa led to outbreaks in a range of species, including coastal seabird populations in Southern Africa [18].

Since 2021, emergence of the current strains of clade 2.3.4.4b virus has resulted in a shift of viral dynamics, affecting a wider range of both avian and mammalian species. The virus has now been detected on all continents, including reaching Oceania for the first time in June 2026 [19], and cases have been reported in over 450 wild bird and mammal species [20]. In Europe, widespread viral transmission during the summer occurred for the first time in 2022, particularly among colony-nesting seabirds [21]. The initial population impacts of this shift were severe, for example, in the UK, HPAI outbreaks in 2021 and 2022 resulted in a 73% decline in great skua (*Stercorarius skua*), a species for which the UK holds an estimated 60% of the global breeding population [22,23]. Further outbreaks caused the loss of over 17% of the Sandwich tern (*Thalasseus sandvicensis*) population in north-western Europe [24] and increased mortality was reported at 75% of Northern gannet (*Morus bassanus*) colonies [25]. The disease continues to spread globally and has been implicated in mass mortality events in South America, with more than 100,000 wild birds reported dead in Peru [26], and subsequent spread from South America to Antarctica [27]. The wide host range of the virus and its ability to spread between continents through bird migration [28], present a growing threat to wild bird populations.

The increased frequency of mass mortality events from HPAI is of substantial concern for conservation of wild bird populations. However, the longer-term implications of outbreaks remain unclear and will depend on the initial population impacts, populations’ ability to recover, and whether there is ongoing mortality through repeated introductions or ongoing circulation of the virus [29]. The population impacts will depend on exposure rates, shaped by species sociality, behaviour, and movement patterns (e.g., migratory and dispersal propensity), as well as the transmissibility of the virus [30] and the composition of the host community [31]. Environmental factors also play a role in transmission [32] and consequently weather patterns and habitat features can influence disease dynamics [33]. Fatality rates following exposure to HPAI will be influenced by immunity from prior exposure to influenza A viruses [34,35], virus pathogenicity, and innate host resistance. Changes to the viral genotype can rapidly change the host range of the virus, as in the case of the white-tailed sea eagle (*Haliaeetus albicilla*) population in Germany, which was not affected in an outbreak of H5N1 in 2006, despite spatial and temporal overlap of mass mortality in waterbirds [36], but experienced a die-off following introduction of an H5N8 strain in the same region in 2016/2017 [37]. This complexity makes prediction of the impacts of an outbreak on a particular population challenging and highlights the need for effective long-term monitoring.

While the scale of the ongoing HPAI panzootic is unprecedented, information on historical outbreaks of this disease, combined with long-term population monitoring data, offers valuable insights into the resilience of wild bird populations to HPAI. In this study, we aim to evaluate the initial and longer-term impacts of HPAI outbreaks on local wild bird populations and to explore how these impacts vary with host and outbreak characteristics. By analysing data from HPAI outbreaks occurring prior to 2020, we infer past population responses to HPAI to provide valuable context for understanding and addressing the current H5 outbreaks, the true extent of which is still being established due to its ongoing global spread, and the need for multiple years of population data to fully understand recovery potential.

## Methods

### Literature search and inclusion criteria

We conducted a comprehensive literature search to identify publications reporting outbreaks of HPAI affecting wild bird populations between 1^st^ January 2000 and 31^st^ December 2020 (S1 Text in S1 File). From this search, we retained all publications which reported an outbreak at a spatially discrete site with clear boundaries (such as a lake), or across a spatially coherent group of sites (such as a coastal region), where HPAI was confirmed as the cause of mortality through PCR detection in carcass swabs. In total, 25 case studies were identified in which HPAI outbreaks occurred, between 2005 and 2018, across Europe, Asia, and Africa. For each of these cases, researchers involved in reporting the outbreak, or monitoring the wild bird populations at the site, were contacted to determine the availability of data on outbreak mortality and population count data for affected species.

Across the 25 case studies, mortality and population-count data were obtained for 76 species-site cases, defined as a single species affected by an outbreak at a single site, involving 39 different species. Of these, 40 cases met the criteria for inclusion in subsequent analyses: (i) a minimum reported mortality of at least 100 individuals, or at least 10 individuals for low-abundance species (such as raptors), species listed as threatened on the IUCN Red List, or at least 1% of the local population and, (ii) a minimum of three years of systematic local population count data available from both before and after the outbreak to quantify population change. For inclusion, population counts needed to have been conducted using a repeatable field methodology that was appropriate for measuring changes in relative abundance for the species and season in question (Table S2 in S1 File). Count data were only considered from periods of the annual cycle when populations were expected to be stable and not influenced by substantial migratory or dispersal movements. Population counts were therefore of either wintering or breeding populations, with the exception of two cases in African penguin where year-round counts were used, due to presence at colonies throughout the year (Table S2 in S1 File; see below).

All selected cases represented disease outbreaks in separate populations, except for outbreaks recorded at a single site, Lake Constance, more than 10 years apart (2005 and 2016). For both outbreaks the same three species met the inclusion criteria and were therefore treated as independent cases. Further details of the literature search methods, selection criteria, and data acquisition are provided in the supporting information (S1 Text in S1 File).

### Data collation

For each species-site case meeting inclusion criteria, in addition to population count and mortality data, we collated additional contextual information from the literature and researchers involved in reporting the outbreak. This included information on viral clade from sequencing (comprising clades 2.2, 2.3.2.1, 2.3.4.4 and 2.3.4.4b), outbreak timing (breeding or non-breeding period) and whether further HPAI disease incidents were detected in years following the initial outbreak (Table S3 in S1 File). As the impacts of HPAI are expected to vary between taxonomic groups due to differences in life history and disease susceptibility, affected species were grouped into six categories: 1) ‘waterfowl’ (Anseriformes), 2) ‘flamingos and grebes’ (Mirandornithes), 3) cranes and rails, hereafter ‘cranes’ (Gruiformes), 4) ‘gulls and terns’ (Laridae), 5) penguins, Suliformes, and pelicans, hereafter ‘seabirds and pelicans’ (Aequornithes), and 6), ‘raptors’ (Table S4 in S1 File).

### Analytical framework and data preparation

#### Overview of analyses conducted.

Changes in population counts provide only an approximate measure of true impacts on local abundance, as counts are affected by detectability and movement of individuals into and out of sites. Nevertheless, analysis of systematically collected, repeated population counts in relation to outbreak timing allows population responses to HPAI to be inferred. We expected HPAI related mortality during outbreaks to cause an initial reduction in local population counts, followed by either recovery (reflected by a positive population trend), or sustained suppression (negative or zero population trend). However, delayed impacts on local populations could also occur from HPAI outbreaks, for example through chronic impacts of infection on reproductive success or additional mortality caused by subsequent outbreaks.

To explore potential impacts of HPAI on populations, we quantified three measures to define outbreak impacts: i) initial change in population count, ii) change in longer-term mean population count and iii) change in population trend (broadly following [38], See Fig S1 in S1 File for a visual summary of methods). By considering these different measures, we identify species-site cases showing evidence of both initial impacts and longer-term declines associated with HPAI. We then conducted analyses to examine correlates of outbreak impact, including taxonomic group and viral clade. In addition, we visualised temporal patterns of change in population counts across taxonomic groups, and extracted a measure of time to recovery for each species-site case.

#### Standardising population counts.

The available population count data varied in the frequency of collection between case studies (See Table S2 in S1 File for details for each case). For case studies with repeated counts per year, these were therefore standardised to an annual estimate to allow for consistent subsequent analysis across all case studies. For these cases, standardised annual abundance estimates were calculated using a negative binomial Generalised Linear Model (GLM). Population count was fitted as the response variable and year and month (both treated as factors), as additive fixed effects. Estimated marginal means for each year were then calculated using the *emmeans* package [39], with estimates averaged over the month effects. These model-based annual estimates represent the expected mean population count for each year after accounting for differences in sampling among months. This approach improves comparability among years by adjusting for uneven or incomplete monthly coverage within years. Population counts for breeding populations recorded as pairs were also converted to individual counts by doubling values, and these standardised annual population counts formed the basis of all subsequent analyses.

### Quantifying outbreak impacts

#### Evaluating initial changes in local population count.

To characterise the initial impact of outbreaks on population counts for each species-site case, we compared counts immediately pre- and post-outbreaks. For most cases (37 out of 40), a population count was conducted during the HPAI outbreak. These counts could not be clearly classified as pre- or post-outbreak and were therefore excluded, meaning initial change was measured over a two-year period. For three cases, no counts coincided with an outbreak, so initial population count change was measured over a one-year period.

For each case, we calculated the percentage change in population count from pre- to post-outbreak as:


$$\begin{array}{c} Initial\;\%\;change\;in\;population\;count=\text{ 100 }\times \;(\frac{N_{post-}N_{pre}}{N_{pre}}) \end{array}$$

Where N_pre_ is the last annual population count pre-outbreak, and N_post_ is the first annual population count post-outbreak. Due to the high level of variation in changes in population counts between cases, for analysis across all cases, initial change was quantified on the log scale as:


$$\begin{array}{c} Log\;change\;in\;population\;count=\text{ log}(\frac{N_{post}+\text{1}}{N_{pre}+\text{1}}) \end{array}$$

The addition of a constant of 1 to the pre- and post-counts was used to avoid infinite values for a single case where the post-outbreak count was 0. Using this scale, a value of 0 indicates no change in population count, negative values indicate a decrease, and positive values an increase.

For species-site cases which showed a decline in the year post-outbreak, we evaluated how closely our estimate of initial population count change aligned with observed mortality. Observed mortality was defined as the total number of carcasses reported in the local population, divided by the larger of the two population counts collected during the outbreak and the preceding year. This approach was used to give a conservative estimate of mortality, accounting for the potential that counts collected during outbreaks may already reflect recent HPAI-related mortality. In a closed population with perfect detectability of both living and dead birds, population change would be expected to directly correlate with observed mortality. In practice, carcass counts are likely to substantially underestimate true mortality as not all carcasses are detected or reported due to limited survey effort and carcass removal, for example by scavengers and tidal movements (e.g., [40]). Population counts are also imperfect measures of abundance and may be influenced by detectability and movement of individuals. In addition, breeding season population counts were typically restricted to breeding pairs, whereas carcass counts may include both adults and juveniles, as age-specific information was rarely available. To quantify the discrepancy between these measures, we modelled the relationship between observed mortality (the total number of carcasses reported as a percentage of the pre-outbreak population count) and the percentage change in population count for declining populations using a linear mixed effects model, with location as a random effect to account for non-independence where multiple species-site cases came from the same site.

#### Evaluating changes in longer-term population count and trend.

Following estimation of the extent of initial change in population counts, we evaluated whether longer-term changes in mean population counts, and population trend occurred. The availability of count data for each species-site case ranged from 3 to 70 years pre-outbreak (median 14 years) and 4–17 years post-outbreak (median 6 years). As a compromise between data adequacy and temporal proximity to the outbreak, analyses of longer-term effects used a time window of up to seven years of counts pre- and post-outbreak, with a minimum of three years of counts (corresponding to the original inclusion criteria).

For each species-site case, the change in mean population counts between pre- and post-outbreak periods was estimated using a GLM, with annual count as the response variable and time period (a two-level factor distinguishing years before and after the outbreak) as the only predictor. Models were fitted with a Poisson error structure and refitted with a quasi-Poisson structure where over-dispersion occurred. The coefficient for time period was exponentiated to generate a proportional change in counts and converted to a percentage change. This method allowed us to estimate the change in mean population count for each species-site case, and the associated standard error.

Change in population trend for each species-site case from pre- to post-outbreak was estimated using a GLM to fit a piecewise regression with annual count as the response variable. A Poisson error structure was initially used, with quasi-Poisson applied when models were over-dispersed. Predictors included year (in this case, a continuous variable estimating the annual linear change in population count or population trend), time period (a two-level factor distinguishing pre- and post-outbreak counts), and their interaction (which tests for a change in the average growth rate, r, between the pre- and post-outbreak periods). Parameter estimates were exponentiated to generate annual population change estimates (r) pre- and post-outbreak. The percentage change in the population trend was calculated as follows:


$$\begin{array}{c} Change\;in\;trend=\text{1}\text{0}\text{0}\;(r_{post-outbreak}-r_{pre-outbreak}) \end{array}$$

#### Analysis of correlates of outbreak impact measures across all cases.

We used linear (mixed) models (fitted using the *lme4* package in R; [41] Bates et al. 2015) to evaluate outbreak impacts across all case studies using our three measures (initial decline, longer-term population count change, and trend change). Initially intercept-only models were fitted, with location as a random effect, to predict a mean change across all cases, and associated 95% confidence interval. To account for variation in the precision of estimates for long-term population count and trend change, species-site cases were weighted by the inverse of the standard error of the change variable (proportional change in population count or trend derived from GLMs).

Following fitting intercept-only models, we then tested for associations between outbreak impacts and the following covariates: taxonomic group (6 levels), viral clade (4 levels), repeated detections of HPAI (2 levels), and initial population count change (continuous). Other potentially relevant covariates, such as outbreak season, habitat association, and species gregariousness, were strongly confounded with taxonomic group and could not be independently evaluated. Observed mortality from carcass counts was not included as a predictor as reporting was highly variable between cases, reflecting the different contexts (e.g., from small, well-monitored sites to large and more remote areas), and therefore population count change was chosen as the measure of initial impact.

For initial population count change, taxonomic group and viral clade were included as fixed effects, with site as a random effect to allow for the non-independence of outbreaks affecting multiple species at the same site. For longer term population count and trend change, taxonomic group, initial count change (continuous) and the occurrence of further HPAI detections during the post-outbreak period (binary) were fitted as fixed effects. Viral clade was not included in the longer-term impacts analysis, on the assumption that any effect of clade would occur through the initial outbreak mortality, which is represented by the initial population count change variable, and any effects through other mechanisms (e.g., due to differential effects of clades on subsequent breeding success) are unlikely to be detectable given the limited sample size. Site was included as a random effect, however, it explained zero variance in the population count change model and produced poor residual fit in the trend model, so it was removed.

Multicollinearity among the explanatory variables was assessed by calculating variance inflation factors (VIF) using the *vif* function from the R *car* package [42]. Residual normality was tested using the *simulateResiduals* function from the *DHARMa* package [43], and the significance of fixed effects was evaluated using the *Anova* function from the *car* package [42]. All analysis was undertaken in R version 4.2.2, with plotting using *ggplot2*.

#### Visualising patterns of population count change between taxonomic groups.

In addition to extracting estimates of change in mean population count and trend, we also visualised the patterns of population counts at a taxonomic group-level for the 10 years pre- and post-outbreak. For each species-site case, we standardised population counts relative to the ‘pre-outbreak baseline’, defined as the last population count prior to the outbreak. The pre-outbreak year was represented as a value of 100, with values greater than 100 indicating an increase relative to the pre-outbreak baseline, while values lower than 100 represent a decrease. For each of the taxonomic groups where there was sufficient data (> 5 case studies), we plotted the median and inter-quartile range of these proportional values for each year relative to the outbreak (where zero is the outbreak year, positive values are post-outbreak and negative values are pre-outbreak).

#### Estimating time to recovery.

For each species-site case, we also derived an approximate estimate of the time taken to recover from outbreak impacts, defined as the number of years until a post-outbreak count was within 5% of the pre-outbreak baseline. Populations were classified as recovered if they reached this baseline within the monitoring period or not recovered if counts remained below the baseline at the end of the monitoring period. It should be noted that failure to reach this baseline may reflect populations which were declining due to other causes, rather than solely impacts of the outbreak.

## Results

### HPAI outbreak characteristics

Across the 25 identified case studies of HPAI outbreaks occurring in wild bird populations pre-2020, there were 76 individual species-site cases (Fig 1). Affected species were from 11 different orders, the majority being Anseriformes (40/76) and Charadriiformes (14/76). Most of the species-site cases were during the non-breeding period (68/76). With the exception of one outbreak caused by an H7 sub-type virus in Algeria, all other outbreaks were caused by H5 viruses of sub-types H5N8, H5N6 and H5N1. Most cases were from the epizootics in 2005–2007 of H5N1 Clade 2.2 (18/76), and in 2016–2018 of H5NX clade 2.3.4.4b (42/76).

**Fig 1:**
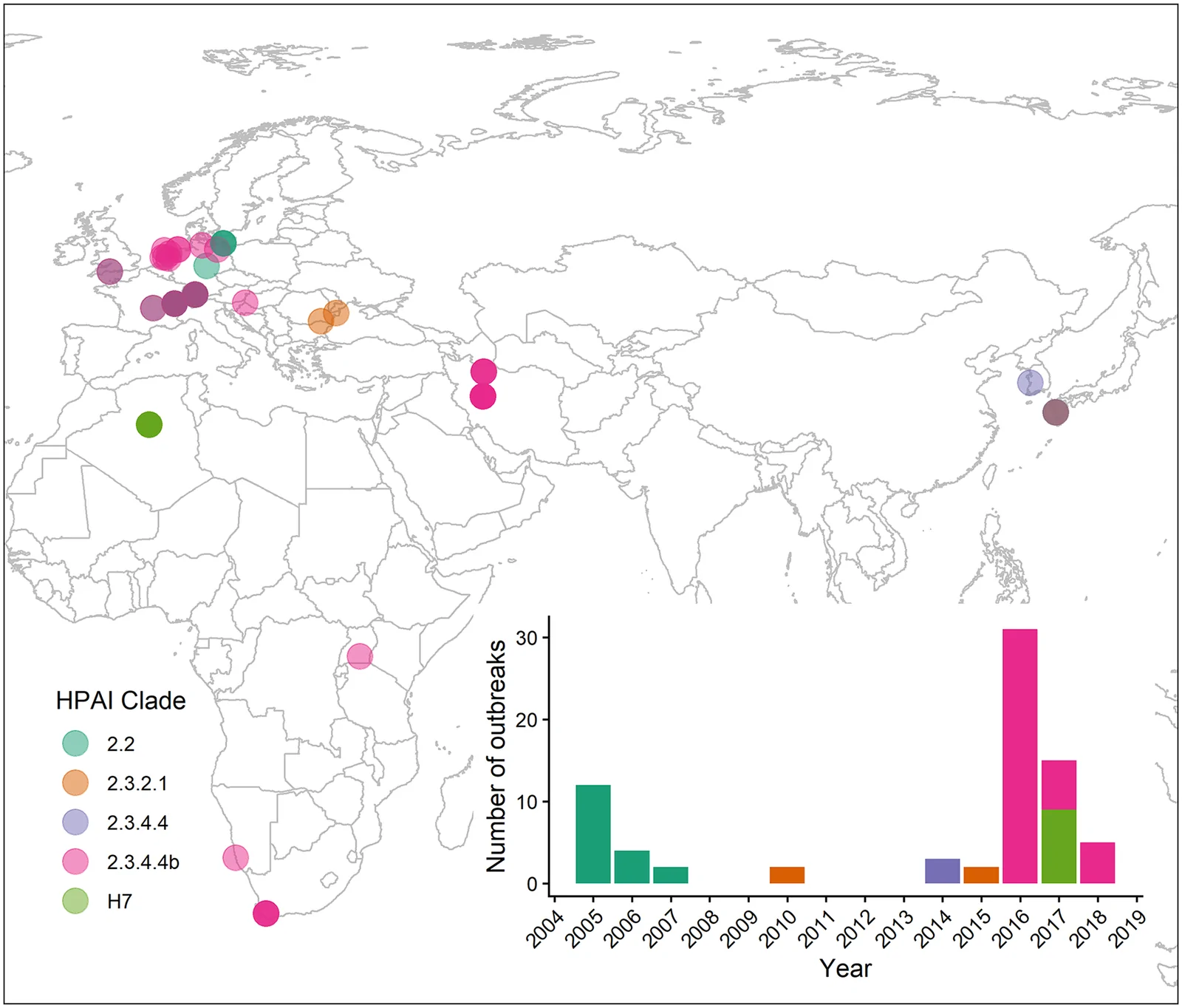
Locations of the 25 identified case studies of HPAI outbreaks in wild bird populations pre-2020 and the viral clade responsible. Outbreak locations are shown alongside a bar plot indicating the year of each outbreak, with colours representing the viral clade. Where multiple species-site cases are included from the same outbreak location, this is indicated by a darker point colour. Map was made using boundaries from geoBoundaries [44] under a CC BY 4.0 license.

All subsequent findings focus on the 40 species-site cases that met the selection criteria, with sufficient count data to estimate longer-term impacts on population count and mortality levels indicative of potential population-level impacts (see methods). A total of 21 of the 40 cases were in Anseriformes (waterfowl), six in Aequornithes (seabirds and pelicans), five in Laridae (gulls and terns), four in Gruiformes (cranes), two in Mirandornithes (flamingos and grebes), and two in raptors. The majority of cases were from clade 2.3.4.4b outbreaks in 2016–2018 (N = 27) followed by 2.2 in 2005–2007 (N = 8), 2.3.2.1 in 2010 and 2015 (N = 4), and one case of 2.3.4.4 in 2014. A total of 31 of the 40 cases were from non-breeding populations, with nine from breeding populations. A description of the individual data sets, and any limitations for inference of HPAI impacts, is presented in Table S2 in S1 File.

### Initial population count changes

The median change in initial population counts following outbreaks across all 40 species-site cases was a 17% decline (IQR of −35% to 5%). In 27 cases, declines occurred in the post-outbreak count, whereas in 13 cases, population counts increased, most likely due to local immigration. For populations which showed a decline in count post-outbreak, the minimum mortality estimate, derived from carcass counts as a proportion of the pre-outbreak population, was not significantly associated with initial population count change (β = 0.13, 95% confidence interval (CI) −0.38 to 0.61, p = 0.61).

Initial population count change varied significantly between taxonomic groups (Fig 2B, *χ*^2^_5_ = 14.3, p = 0.01). Predicted initial declines (estimated marginal means for log population change) were greatest amongst gulls and terns (−1.59, 95% CI −3.04 to −0.13%). Mean values also suggested initial declines in flamingos and grebes (−0.12, 95% CI −2.04 to 1.79%), and seabirds and pelicans (−0.02, 95% CI −1.46 to 1.43%) (Fig 2B). However, the wide confidence intervals reflect marked variation in initial changes within taxonomic groups, with substantial declines evident for at least one species in each taxonomic group. Initial declines exceeding 20% were evident for 80% of gulls and terns (4/5), 50% of seabirds and pelicans (3/6), 50% of flamingos and grebes (1/2), 50% of raptors (1/2), 43% of waterfowl (9/21) and 25% of cranes (1/4). Initial population count changes did not differ significantly between viral clades (Fig 2C, *χ*^2^_3_ = 4.3, p = 0.23).

**Fig 2:**
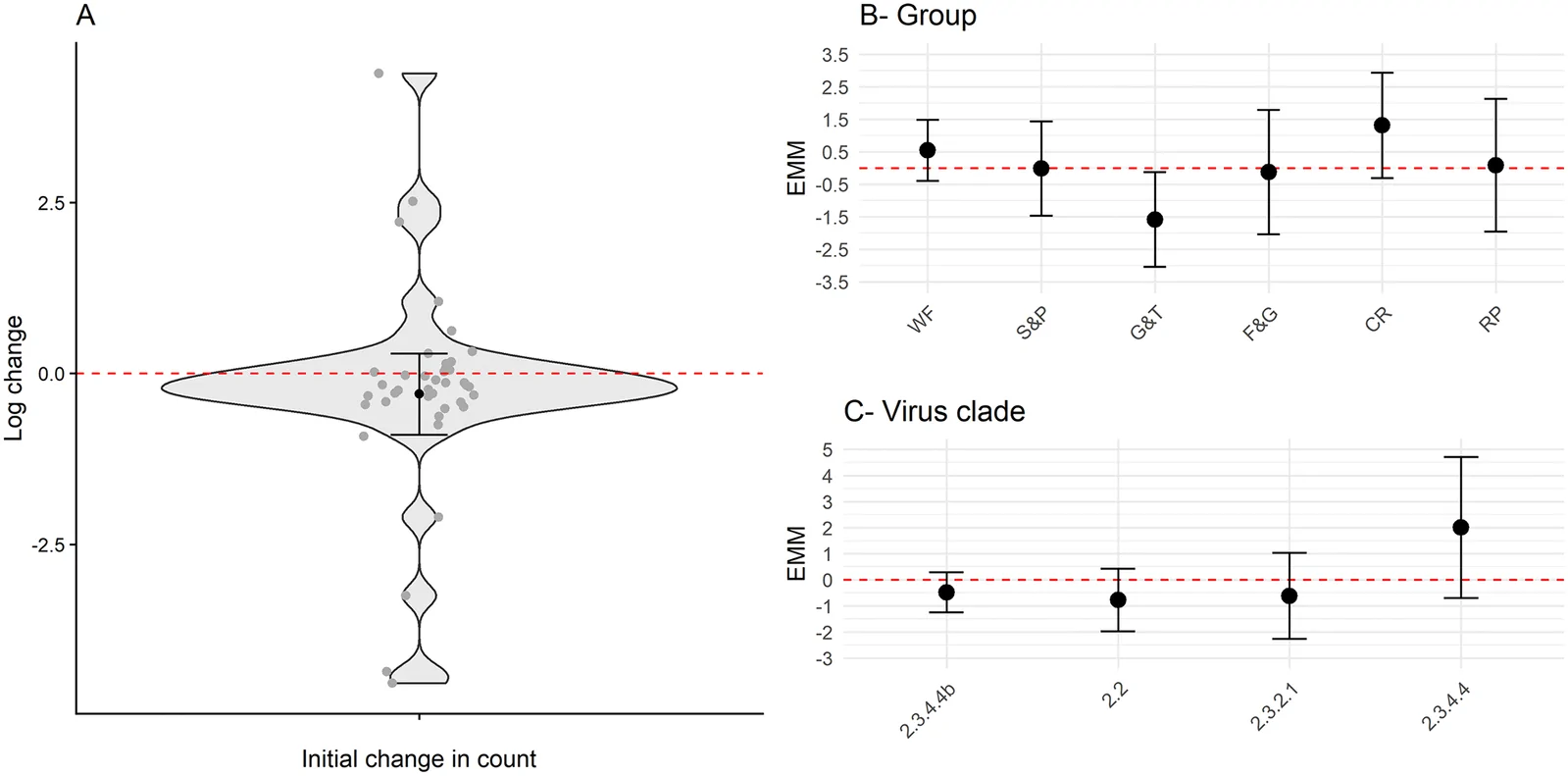
Initial impacts of HPAI outbreaks on local population counts. A) Distribution of initial local population count change following HPAI outbreaks, shown on the log scale. Grey points show the estimate for individual species-site cases. The black point and error bars show predicted mean change (estimated marginal mean) and 95% confidence interval across all cases. The red dotted line indicates no change in population count. Panels B and C show predicted values (estimated marginal means (EMM) shown as black points) from the mixed effect model for species groups and virus clade respectively, with error bars representing 95% confidence intervals. Species groups: WF- waterfowl, S&P- seabirds and pelicans, G&T- gulls and terns, F&G- flamingos and grebes, CR- cranes and rails, RP- raptors.

### Longer-term population changes

Across all 40 cases, 20% (8/40) exhibited a significant decline in longer-term mean population count post-outbreak, while 18% (7/40) showed a significant increase (Table S5 in S2 File). Considering the mean change across all cases, there was no evidence of decline (Fig 3A, intercept-only GLM, weighted by standard error of change for each case: 1.8% increase, 95% CI: −12% to +18%, p = 0.80). The analysis of correlates for this measure showed no evidence of an association with the initial population count change (F_1,32_ = 0.19, p = 0.66), taxonomic group (F_5,32_ = 0.69, p = 0.63) or the occurrence of further HPAI detections (F_1,32_ = 1.02, p = 0.31).

**Fig 3:**
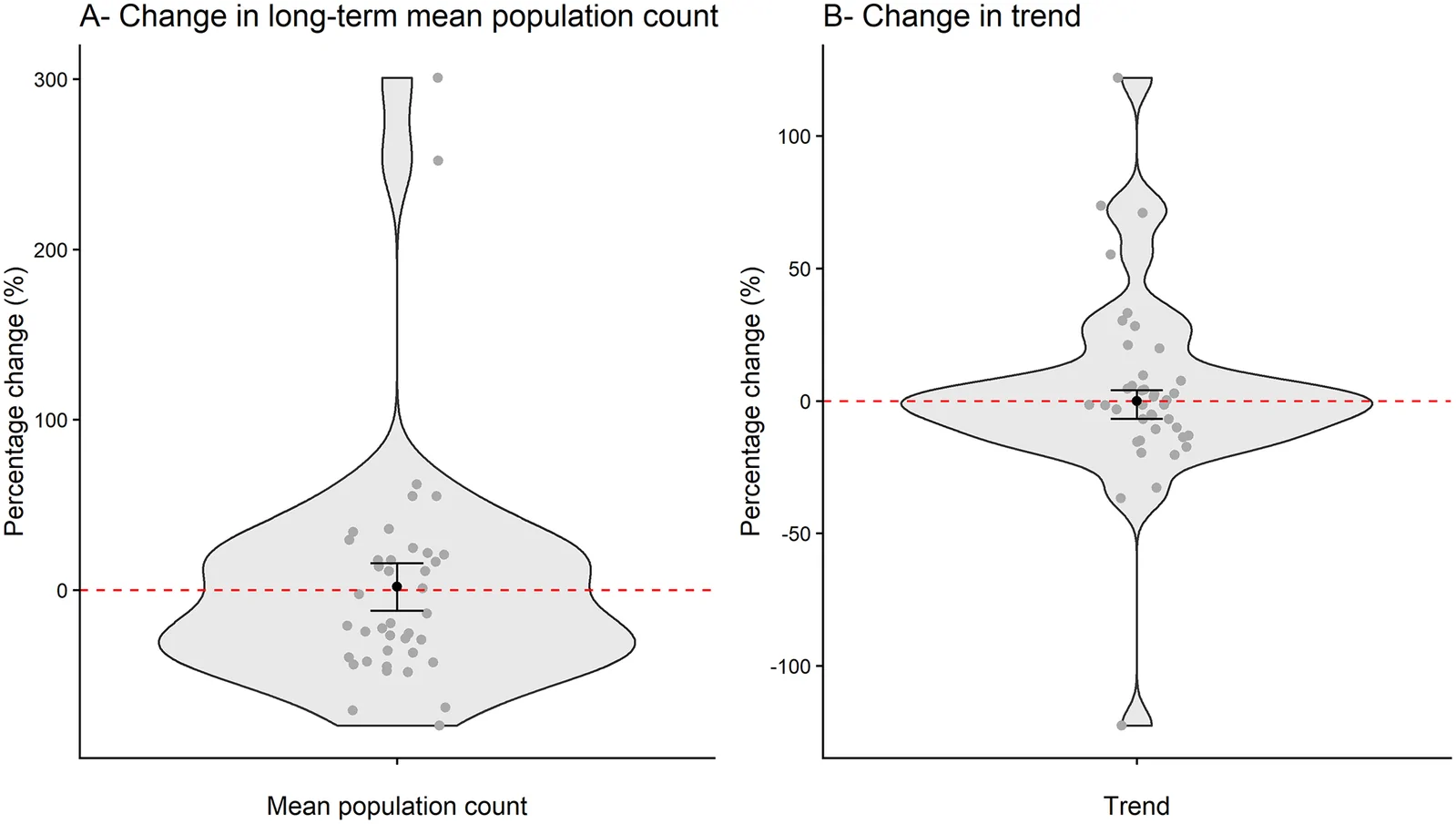
Distribution of longer-term changes in A) mean population count and B) population trend before and after HPAI outbreaks. The red horizontal line indicates no change in the estimated measure while grey points show the estimates for individual species-site cases. Black points and error bars show predicted mean changes, and 95% confidence intervals, across all cases, from the intercept-only generalised linear model in which values were weighted by the inverse of the standard error.

Prior to HPAI outbreaks, most of the local populations (68%, 27/40) exhibited positive population trends with a median trend of 3% growth per annum (IQR: −2% to +9%). Following the outbreak, 18 of the 40 cases (45%) showed positive population trends, and 22 negative trends, with a median trend of a 2% decline per annum (IQR: −5% to +7%). Two cases showed a significant decrease in population trend, and four cases a significant increase (Table S5 in S2 File). However, the mean change in population trend across all cases did not differ significantly from zero (Fig 3B, intercept only GLM, with observations weighted by the inverse of the standard error of the change: −1.3%, 95% CI: −6.7 to +4.1%, p = 0.62). The correlates analysis found no evidence that changes in trend were associated with initial population count change (F_1,32_ = 3.78, p = 0.06), taxonomic group (F_5,32_ = 0.95, p = 0.46), or the occurrence of repeated HPAI detections (F_1,32_ = 0.23, p = 0.63). Across all cases, longer-term changes in population count and trend were uncorrelated (r_s_ = −0.12, CI −0.41 to 0.20).

Although no significant differences between taxonomic groups were found for change in long-term mean count or trend, visualisation of the relative population counts pre- and post- HPAI outbreaks for taxonomic groups with larger sample sizes (N > 5) did indicate variation in temporal patterns of decline and recovery (Fig 4). Waterfowl (21 cases) showed no consistent change in counts following outbreaks, while seabirds and pelicans (6 cases), and gulls and terns (5 cases) showed more consistent declines, with population averages remaining below the pre-outbreak baseline for 4 and 2 years respectively. For flamingos and grebes, cranes, and raptors, the number of available species-site cases was too small (less than 5) for general patterns to be inferred from summaries of population counts.

**Fig 4:**
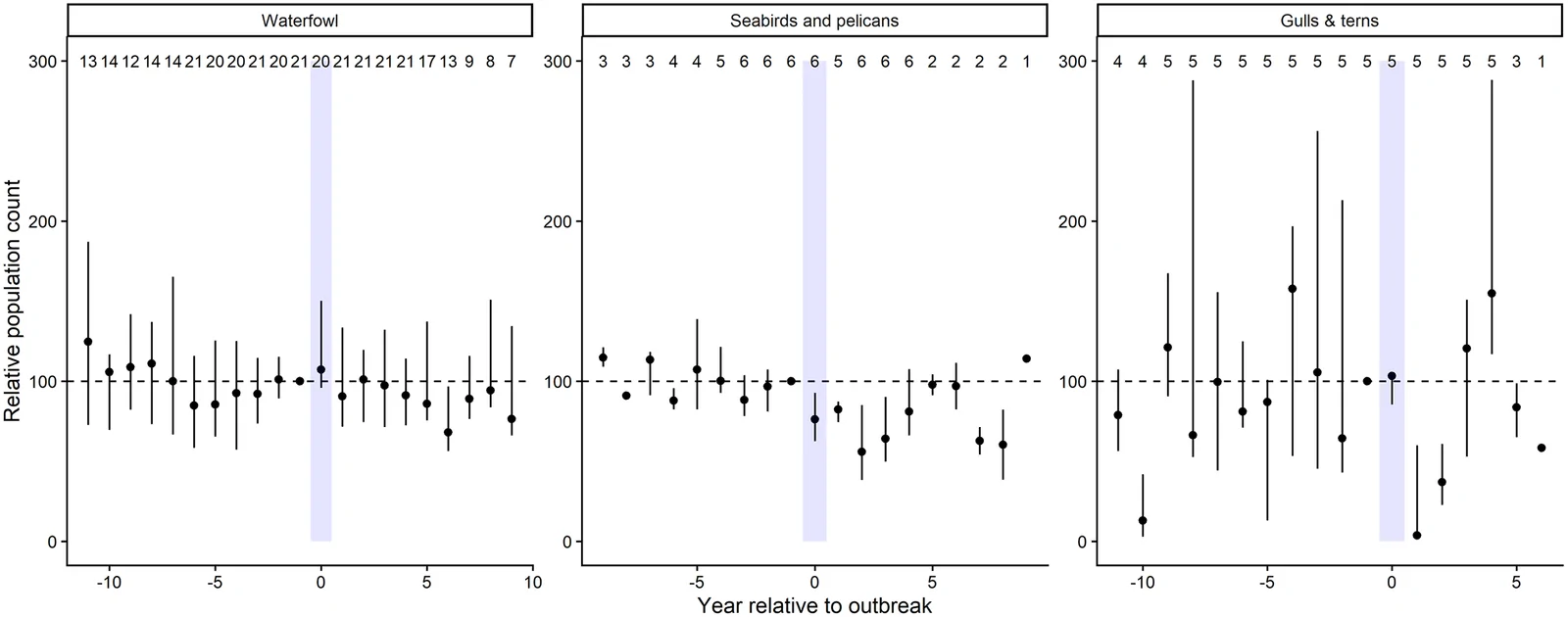
Average changes in relative population counts of Waterfowl (Anseriformes), Seabirds and pelicans (Aequornithes), and Gulls & terns (Laridae) before and after HPAI outbreaks. For each species-site case, annual counts were divided by the final pre-outbreak count and multiplied by 100 to generate relative population counts. A value of 100 therefore indicates no change in population count post-outbreak, while a value of 90 represents a 10% reduction. Points show the median values and error bars interquartile ranges for each taxonomic group. Year 0 represents the outbreak year, indicated by vertical blue shading with negative/positive values showing pre- and post-outbreak years respectively. The number of species-site cases contributing data in each year are shown above the points.

Of the 27 cases which exhibited initial declines in population count following the outbreak, 17 took a median of 5 years (range 3–8 years) to recover to within 5% of the pre-outbreak baseline, and 10 failed to recover by the end of the monitoring period (4–17 years after the initial outbreak).

Declines across all three measures of outbreak impact (initial population count change, longer-term change in average population count and population trend), indicating that declines occurred during the outbreak and persisted afterwards, were observed in eleven cases across seven species, although not all changes were statistically significant (Table 1). With the exception of a single case (mallard, Lake Constance), these species-site cases were the same as those identified as having not recovered to the pre-outbreak baseline within the monitoring period.

**Table 1:** Summary of species-site cases where decline occurred across all three impact measures. The names of threatened species on the IUCN red list are indicated in bold. Significant changes in trend or longer-term population count are indicated in bold.

Species	Location & outbreak year	Initial change (%)	Longer-term change in population count (*p* value)	Change in trend (*p* value)	Years post outbreak to recovery within 5% of baseline (available years of data post-outbreak)
**African penguin**	Halifax island, Namibia. 2019.	−47.0	**−42.6% (p < 0.01)**	−13.1% (p = 0.12)	No recovery (4 years)
	Western and Eastern Cape provinces, South Africa. 2017.	−17.6	**−39.7% (p < 0.01)**	−7.0% (p = 0.06)	No recovery (4 years)
**Cape cormorant**	Western and Eastern Cape provinces, South Africa. 2017.	−25.5	−19.6% (p = 0.36)	−10.8% (p = 0.55)	No recovery (4 years)
**Common pochard**	Lake Constance, Germany, Austria, and Switzerland. 2005.	−60.3	**−37.0% (p = 0.02)**	−13.7% (p = 0.11)	No recovery (17 years)
Great black-backed gull	Wadden Sea, Netherlands. 2016.	−40.1	−24.5% (p = 0.21)	−17.3% (p = 0.21)	No recovery (4 years)
Mallard	Lake Constance, Germany, Austria, and Switzerland. 2005.	−12.7	−2.5% (p = 0.78)	−7.0% (p = 0.33)	5 years
Peregrine falcon	Wadden Sea, Netherlands, 2016.	−28.4	**−21.1% (p = 0.02)**	−1.4% (p = 0.85)	No recovery (4 years)
Tufted duck	Lake Constance, Germany, Austria, and Switzerland. 2016.	−9.5	**−42.2% (p < 0.01)**	−1.5% (p = 0.82)	No recovery (6 years)
	Lake Plon, Germany. 2016.	−52.9	−25.6% (p = 0.26)	**−36.8% (p < 0.01)**	No recovery (7 years)
	North Holland and Flevoland, Netherlands. 2016.	−21.2	**−45.0% (p < 0.01)**	−3.3% (p = 0.82)	No recovery (4 years)
	Gelderland and Flevoland, Netherlands. 2016.	−28.4	−13.9% (p = 0.41)	−15.1% (p = 0.27)	No recovery (4 years)

These eleven cases included seabirds (African penguin (*Spheniscus demersus*)*,* Cape cormorant (*Phalacrocorax capensis*)), waterfowl (common pochard (*Aythya ferina*), mallard (*Anas platyrhynchos*), and tufted duck (*Aythya fuligula*)), a gull species (great black-backed gull (*Larus marinus*)) and a raptor (peregrine falcon (*Falco peregrinus*)). Notably, African penguin has recently been uplisted to globally Critically Endangered on the IUCN Red List, while Cape cormorant is classified as Endangered, and common pochard as Vulnerable.

## Discussion

Our analysis of local population changes following historical outbreaks of HPAI, occurring pre-2020 (2006–2018), demonstrates that this disease can have substantial impacts on wild birds, with an average initial decline in population counts of 17% following mass-mortality events. In eleven of the 40 cases for which suitable data were available, initial declines in count occurred and were followed by longer-term reductions in both mean count and trend, indicating prolonged negative consequences for these populations. While we cannot exclude other causes of population change, such as immigration or emigration from wintering populations, by combining data from 40 case studies, this study provides an indication of the impacts across wild bird populations from these historical outbreaks. These longer-term impacts affected a variety of taxonomic groups including seabirds and waterfowl, as well as a gull and a raptor. However, for the majority of affected populations (30/40), counts recovered to within 5% of pre-outbreak baselines within the monitoring period. Notably, there have been key shifts in the dynamics of HPAI since 2020, with an increase in the geographic and species range affected, and significant outbreaks in breeding seabird colonies, which may result in more severe long-term impacts relative to the cases considered here.

### Taxonomic variation in HPAI-related population impacts

#### Seabirds, gulls and terns.

Initial impacts of HPAI on population counts were particularly apparent for cases in Laridae (gull and tern) species, and to a lesser extent Aequornithes (seabirds and pelicans). The declines in these taxa are consistent with the impacts of H5 clade 2.3.4.4b virus documented globally in more recent outbreaks since 2021 [23,24,26,45]. The life-histories of these groups make them vulnerable to disease outbreaks, as they typically breed in dense colonies that are interconnected through dispersal [46]. Of the cases considered here in seabirds (Aequornithes), all six outbreaks were in the breeding period. However, in the gulls and terns (Laridae species), mortality in some species also occurred during the non-breeding period. This included a great black-backed gull population in the Netherlands, which underwent long-term declines, and for which exposure was likely to have been through scavenging on carcasses [47,48], and a winter aggregation of white-winged tern at Lake Victoria, Uganda. Recovery of these populations is also expected to be slower due to these being long-lived species with delayed breeding. While initial declines may be less apparent due to younger individuals moving into vacant spaces in colonies and starting to breed earlier, an increase in inexperienced individuals may impact colony productivity, hindering population recovery [23,49]. Following initial declines some populations such as Cape gannet (*Morus capensis*), Pallas’s gull (*Ichthyaetus ichthyaetus*), swift tern (*Thalasseus bergii*), and white-winged tern (*Chlidonias leucopterus*), recovered to pre-outbreak levels whilst others such as, great black-backed gull, African penguin, and Cape cormorant continued to decline post-outbreak. It should be noted that other causes may have contributed to the observed mortality in Cape cormorant and Cape gannet populations [50]. Declines in African penguin have also been driven by multiple causes, including marine noise pollution, competition with the fishing industry, environmental fluctuations related to climate change, and extreme weather events [50–53]. HPAI, however, is an additional mechanism that is likely to exacerbate these declines and limit recovery, and further outbreaks have occurred in these populations since 2020.

#### Waterfowl.

Most cases in waterfowl showed no significant change in population count following HPAI outbreaks, despite mass-mortality events. Waterfowl are primary hosts of avian influenza viruses and often carry the disease with minimal mortality, likely related to their lower susceptibility to influenza A viruses and cross-immunity from prior exposure to low pathogenicity strains [54]. Our data on Baikal Teal (*Anas formosa*) highlight this apparently low susceptibility with an estimated 200 fatalities from a local population of more than 300,000, despite this species forming large dense flocks at the time of the outbreak. Even where significant mortality does occur, the faster life history of waterfowl relative to seabirds also allows more rapid recovery. For example, following an outbreak of HPAI in Barnacle Geese (*Branta leucopsis*) on the Solway Firth, UK in the winter of 2021/22 where approximately 31% of the population succumbed, high breeding productivity in the subsequent two years led to complete recovery in population counts [40]. However, a subset of waterfowl cases in our study did entail sustained declines, most notably tufted duck in four different cases (Table 1), confirming the high susceptibility to HPAI for this species noted previously [55]. Longer-term changes in waterfowl populations may also be driven by other factors. For example, in migratory populations ‘short-stopping’ may be occurring, whereby a shift occurs in wintering distributions, due to factors such as climate or changes to food distribution [56].

#### Cranes, flamingos and grebes.

Population counts of three of the four species in the ‘cranes’ group (common coot (*Fulica atra*), hooded crane (*Grus monacha*), white-naped crane (*Antigone vipio*)) increased following HPAI outbreaks. Although common crane at the Mighan wetland (Iran) showed a large initial decline and the post-outbreak population count was significantly reduced, the species was declining prior to the HPAI outbreak, and the post-outbreak trend was less negative than before the outbreak. The role of HPAI in causing this decline is therefore unclear. More recent HPAI outbreaks have documented large disease-related mortality amongst common crane (*Grus grus*) suggesting high susceptibility to the more recently circulating viral strains [57]. Despite evidence of substantial initial declines, neither species in the ‘flamingo & grebe’ group exhibited longer-term HPAI-related declines: post-outbreak population counts of greater flamingo (*Phoenicopterus roseus*) increased, while the breeding black-necked grebe (*Podiceps nigricollis*) population initially declined following HPAI and subsequently recovered to pre-outbreak numbers before going locally extinct, potentially due to on-site water level change (Fischer, S. Personal Communication).

#### Raptors.

One of the two raptor populations (peregrine falcon wintering in the Dutch region of the Wadden Sea) experienced a sustained post-outbreak decline in population counts. Exposure to HPAI in peregrine falcons is most likely to occur while hunting infected waterfowl and waders [47,48]. HPAI outbreaks in raptors may be under-recorded as these species often occur at low densities, and unusual levels of mortality are less obvious than in large aggregations of individuals. However, impacts of HPAI on breeding success and adult mortality in raptors have been documented since 2021 [58–62]. The other raptor species, white-tailed eagle, showed no discernible post-outbreak change in population count. Mortality primarily affected juveniles and chicks [37], and the absence of impact on breeding numbers may reflect the delayed age at first breeding (typically 5 years) and/or the relatively low sensitivity of population growth to breeding success and juvenile survival in long-lived species [63].

### Effects of viral clade and repeated outbreaks on population impacts

Our data did not suggest significant differences in population-level impacts of outbreaks caused by different clades of H5 HPAI. However, as we only considered outbreaks where a minimum level of mortality occurred, this precluded consideration of populations in which HPAI may circulate with lower, or no, associated mortality. Furthermore, we had limited numbers of cases for each clade, and a larger data set would be needed to characterise how population impacts vary depending on the specific interactions between viral clades and the host species infected. Even within viral clades, substantial variation in virus pathogenicity and host specificity can occur between different genotypes, as seen with the emergence of the gull-adapted BB genotype in 2023. which caused significant impacts on black-headed gull (*Chroicocephalus ridibundus*) populations, which were relatively unaffected in the previous year despite there also being widespread circulation of other clade 2.3.4.4b strains [63]. With the limited data available, we were unable to explore these genotype-level effects and interaction with species group. Our data mainly comprised outbreaks from the clade 2.3.4.4b epizootic in 2016–2018 and from the clade 2.2 epizootic in 2005–2006. The former is highly transmissible in wild birds [12], with a broad host and geographic range, which increases the potential for viral mutation and recombination, which can lead to increased virulence and mortality [64]. Clades 2.3.2.1 and 2.3.4.4 showed more limited geographic spread. From our case studies, clades 2.3.2.1 viruses caused the outbreaks in Balkan breeding Dalmatian pelicans, and hooded and white-naped cranes in Japan and South Korea, while 2.3.4.4 was evident only in wintering Baikal teal. Notably, although we only had access to population count change data relating to H5 outbreaks, mass wild bird mortality has been recorded for other HPAI sub-types. For example, more than a thousand wild bird fatalities were reported from an H7 outbreak in Sabkhat el Malah, Algeria, and H7 sub-types have recently spread in Australia and South Africa [65].

The detection of additional HPAI cases following the initial disease outbreak had no detectable effect on the population count changes of the host species in our study. This finding is consistent with several studies showing the development of acquired immunity in surviving individuals following HPAI outbreaks, which may buffer the impacts of further outbreaks [25,66,67]. For example, in the mute swan (*Cygnus olor)* population at Abbotsbury swannery in the UK, seroprevalence against influenza A viruses increased with age, and older individuals experienced lower mortality, suggesting that baseline flock immunity can mitigate the effects of repeated HPAI incursions [68]. Multiple cases in this study involved populations that were stable or increasing despite repeated HPAI detections in the period following disease outbreaks [37,68,69], suggesting repeated exposure to HPAI does not threaten local population status. On the other hand, populations having only sporadic exposure and low baseline immunity to Influenza A viruses may be more vulnerable to mass mortality events. Further research is required to improve understanding of the persistence of immunity following HPAI outbreaks in wild bird populations, and how this influences population recovery.

### Study limitations

Inferring impacts from population counts has limitations, as the local scale of most of these counts means they are subject to variation caused by local immigration or emigration. The majority of our cases were wintering populations (31 of 40), whereas only nine were from breeding populations. Cases in breeding populations are expected to be less affected by movements as most birds show high fidelity to breeding locations in successive years. By contrast, winter bird counts may vary in relation to fluctuating local site conditions (e.g., variable rainfall affecting wetland water levels) or may be subject to longer-term trends, for example due to short-stopping driven by climate [70]. We were also unable to account for differences in detection probabilities between species, sites and years, which may affect both the population count estimates and carcass counts. The limitations of the available data were highlighted by the lack of correlation between observed minimum mortality from carcass counts and initial population count change as measures of outbreak impact. The reliability of carcass counts will vary between species and sites, depending on the likelihood that bird carcasses will be detected (e.g., via variation in human search effort or vegetation structure) and the fates of carcasses (e.g., via scavenger density or tidal movements). While carcass counts can be a valuable means of identifying unusual mortality patterns, combining these with population monitoring is therefore needed to characterise the extent of impacts. It should be noted that some of our case populations were also supported by human interventions, such as supplementary feeding of cranes in Japan, or nest platforms provided for Dalmatian pelicans [71] which might affect local dispersal patterns. Our analyses minimise the potential impacts of these potential data limitations by testing for longer-term population count changes that allow for between-year fluctuations and any pre-existing trends in population count.

### Implications for response to ongoing outbreaks

Our results highlight the importance, but also the challenges, of characterising the impacts of ongoing outbreaks of HPAI on wild bird populations. Availability of baseline population data was key to this study, and to facilitate future research on HPAI impacts should be collected for any populations considered at high risk of outbreaks. When outbreaks occur, we encourage collation of mortality data (carcass counts) from all species. Even if only approximate estimates are possible, this allows identification of potentially affected species, where follow-up population monitoring should therefore be prioritised. To reliably identify impacts from population counts requires systematically collected data at a scale relevant to the outbreaks. For example, annually collected international waterbird census (IWC) data was a key source of information in this study. To understand the full impacts, continued monitoring for multiple years is required, as the full effect of outbreaks may take time to be visible. For example, for slow-lived seabird species where breeding is delayed, it may take multiple years before the impacts of outbreak years on lost breeding success are fully observed in population counts. For wintering populations which are highly mobile, effects of disease may be challenging to distinguish from drivers of immigration or emigration to local sites. Systematic counts across multiple sites may therefore be needed to identify the full picture, and ideally multiple counts per season to increase the reliability of population estimates.

The breadth of avian species affected by HPAI, as shown in this study by cases in waterfowl, seabirds, cranes, grebes, and raptors, shows that comprehensive monitoring is required to understand the full impacts. Overall, our results highlight the need for effective integration of disease surveillance, and systematic population monitoring, to fully understand the population-level consequences of outbreaks. This information can be used to prioritise conservation action to recover those species most at risk.

### Conclusions

Our study found that following HPAI outbreaks prior to 2020, the majority of populations showed limited impacts, or recovered following initial declines. However, in a quarter of cases, initial declines were not recovered within the monitoring period (10/40 comprising mainly seabirds and waterfowl and including three globally threatened species). The magnitude of impacts was variable even amongst closely related species sharing similar ecological and life history traits highlighting the challenges in predicting population-level impacts. Continued monitoring of affected populations is essential to assess the impacts of the ongoing global spread of HPAI on wild birds globally, and to prioritise responses to reduce impacts, and promote recovery for the most vulnerable species.

## Supporting information

S1 File Contains details of literature search methods (S1 Text, Table S1), descriptions of HPAI outbreaks and associated mortality and count data (Table S2), descriptions of virus and host-species traits (Table S3), species groupings used in analyses (Table S4) and a visual summary of the outbreak impact measures (Fig S1). (DOCX)

S2 File Contains supplementary results (S2 Text) including descriptions of carcass counts and observed mortality (Fig S2), summary abundance changes for each case (Table S5), the sensitivity of predicted changes in count (Fig S3) and trend (Fig S4) to the number of post-outbreak years included in the assessment. Also contains residual plots to evaluate model assumptions for initial change in count (Fig S5), longer-term change in count (Fig S6) and longer-term trends (Fig S8), as well as model effect sizes for longer-term count changes (Fig S7) and trends (Fig S9).
(DOCX)
