# Supplementary material for: Characterising the longer-term impacts of highly pathogenic avian influenza outbreaks prior to 2020 on wild bird populations
Source: PLoS One. 2026 Nov 11;21(11):e0360235. doi: 10.1371/journal.pone.0360235 (PMC13754095; doi:10.1371/journal.pone.0360235)
Supplement: Supplementary file 1 [file pone.0360235.s001.docx]

**Supporting Information**

**Characterising the longer-term impacts of highly pathogenic avian influenza outbreaks prior to 2020 on wild bird populations**

**S1 Text: Literature search methods**

We searched the literature using Google Scholar (https://scholar.google.co.uk), ISI Web of Science (https://www.webofknowledge.com) and ProQuest (https://www.proquest.com), using the term; ‘wild bird*’, alongside a term relevant to a HPAI outbreak (one of: Highly Pathogenic Avian Influenza, Avian Influenza, HPAI, H5N1, or H5N8), and an associated cost (mortality, or Population). We also included all studies cited within several key articles (Olsen et al. 2006, Gauthier-Clerc et al. 2007, Kleyheeg et al. 2017, Lycett et al. 2019) and performed a forward search to include any study citing these. Searches were conducted for all literature published from 1st January 2000, including peer-reviewed literature, but also non-peer reviewed reports and web articles, as the results of state or government agency testing for HPAI is readily accessible online. As such, we accessed the World Organisation for Animal Health database (WAHIS https://wahis.woah.org), and the Food and Agriculture Organisation of the United Nations (FAO) Global Animal Disease Information System (https://empres-i.apps.fao.org/) to identify further outbreaks of HPAI, including less-known outbreaks, or those in remote locations.

***Table S1****-* ***Details of search methods to identify HPAI outbreaks pre-2020.*** *The search engines and terms used to identify literature focused on HPAI outbreaks in wild bird populations, including the number of hits each search provided. All hits were screened unless the search provided more than 2,500 hits.*

| **Search engine** | **Search terms** | **Date** | **Number of hits** | **Proportion screened** |
| --- | --- | --- | --- | --- |
| **Scholar** | ("Highly Pathogenic Avian Influenza" OR "HPAI" OR "H5N1" OR "H5N8") AND "Wild bird" AND ("mortality" OR "population") | 27/10/2022 | 7100 | 35% |
|  | *Within those citing Olsen et al. 2006*: ("Highly Pathogenic Avian Influenza" OR "HPAI") AND "Wild bird" AND ("mortality" OR "population") | 27/10/2022 | 1090 | 100% |
|  | *Within those citing Gauthier-Clerc et al. 2007*: ("Highly Pathogenic Avian Influenza" OR "HPAI") AND "Wild bird" AND ("mortality" OR "population") | 27/10/2022 | 152 | 100% |
|  | *Within those citing Lycett et al. 2019*: ("Highly Pathogenic Avian Influenza" OR "HPAI") AND "Wild bird" AND ("mortality" OR "population") | 27/10/2022 | 59 | 100% |
|  | *Within those citing Kleyheeg et al. 2017*: ("Highly Pathogenic Avian Influenza" OR "HPAI") AND "Wild bird" AND ("mortality" OR "population") | 27/10/2022 | 60 | 100% |
| **ISI Web of Science** | ("Highly Pathogenic Avian Influenza" OR "HPAI" OR "H5N1" OR "H5N8") AND "Wild bird" AND ("mortality" OR "population") | 31/10/2022 | 105 | 100% |
|  | "Avian influenza" AND "Wild bird" AND "Mortality" | 31/10/2022 | 106 | 100% |
|  | "Highly Pathogenic Avian Influenza" AND "Wild bird" | 31/10/2022 | 456 | 100% |
| **ProQuest** | ("Highly Pathogenic Avian Influenza" OR "HPAI" OR "H5N1" OR "H5N8") AND "Wild bird" AND ("mortality" OR "population") | 11/11/2022 | 3056 | 82% |

The search identified over 7,000 publications, reports and webpages. Studies were retained that satisfied all four of the following criteria: (1) at least one wild bird tested clinically positive for HPAI (any strain of H5 or H7 HPAI), excluding captive and captive-bred individuals, but including species non-native in range, (2) a concurrent increase in mortality likely related to HPAI, with a minimum of 100 or more individuals from any single wild bird species reported dead. A lower mortality threshold of 10 individuals was applied for less abundant species such as birds of prey, or globally rare species (defined as IUCN status Near Threatened, Threatened, Endangered or Critically Endangered), (3) the outbreak was reported at a defined site (with clear boundaries such as a lake), or a geographically restricted group of sites (such as a coastal region), for which surveillance of local avian population counts and mortality was feasible (i.e. excluding reports describing outbreaks extending over broad geographic regions), and (4) outbreaks occurred between 1^st^ January 2000 and 31^st^ December 2020 as it was deemed necessary to have at least three years of follow up population monitoring to assess the potential longer-term impacts of the outbreak on local population size. Under these inclusion criteria outbreaks in 25 different locations were identified worldwide (Table S2), which became the focus of a data requisition process.

**Table S2- Description of outbreaks included and data sources.** Estimated mortality for all wild bird species during each outbreak is presented based on observed carcasses. For species involved, all species where mortality was observed and data available are shown. Species which met inclusion requirements for analysis are presented in bold. Estimated mortality by species is provided in the open access data. Abbreviations: IWC, international waterbird census count. WBS, Waterways Bird survey. WAHIS, World Animal Health Information System.

| **Location** | **Year** | **Season** | **Sub-type** | **Species** (Species in bold met inclusion criteria for analyses) | **Mortality across all species**  (Source) | **Field survey method**  (Years covered) | **Comments on data quality for interpretation of HPAI impacts** |
| --- | --- | --- | --- | --- | --- | --- | --- |
| **Netherlands,** Friesland | 2016 - 2017 | Winter | H5N8 | **Eurasian Wigeon** | >13,500  (SOVON Dutch Centre for Field Ornithology) | IWC winter counts. For Peregrine falcon, the seasonal maximum of monthly counts was used. For other species, the seasonal average of monthly counts was used.  (2010 – 2020). | **Frequency of counts:** Monthly counts  **Effects of bird movement:** Wintering populations, therefore potential for inter-annual variation due to immigration and emigration |
| **Netherlands,** Noord-Holland | 2016 - 2017 | Winter | H5N8 | **Eurasian Wigeon** |  |  |  |
| **Netherlands,** N2000 Wadden Sea | 2016 - 2017 | Winter | H5N8 | **Great Black-backed Gull, Peregrine Falcon** |  |  |  |
| **Netherlands,** N2000 Markermeer & IJmeer | 2016 - 2017 | Winter | H5N8 | **Tufted Duck** |  |  |  |
| **Netherlands,** N2000 North Sea | 2016 - 2017 | Winter | H5N8 | **Great Black-backed Gull** |  |  |  |
| **Netherlands,**  N2000 Veluwerandmeren | 2016 - 2017 | Winter | H5N8 | **Tufted Duck** |  |  |  |
| **Germany,** Schleswig-Holstein, Großer Plöner See. | 2016 | Winter | H5N8 | **Tufted Duck** | >250  (Ornithological working group for Schleswig-Holstein and Hamburg) | Monthly IWC winter counts  (2000 - 2020) | **Frequency of counts:** Monthly counts  **Effects of bird movement:** Wintering populations, therefore potential for inter-annual variation due to immigration and emigration |
| **Germany,** Lake Constance | 2005 | Winter | H5N1 | **Tufted Duck**, **Common Pochard,**  **Mallard**, **Mute Swan**,  Black-headed Gull, | >600  (Ornithologische Arbeitsgemeinschaft Bodensee) | Mean count from 8 monthly IWC winter counts.  (2000 - 2020) | **Frequency of counts:** Monthly counts  **Effects of bird movement:** Wintering populations, therefore potential for inter-annual variation due to immigration and emigration |
|  | 2016 | Winter | H5N8 | **Tufted Duck,** **Common Pochard,**  Mallard**, Mute Swan**,  Black-headed Gull, | ~767  (Ornithologische Arbeitsgemeinschaft Bodensee) | Mean count from 8 monthly IWC winter counts.  (2000 - 2020) | **Frequency of counts:** Monthly counts  **Effects of bird movement:** Wintering populations, therefore potential for inter-annual variation due to immigration and emigration |
| **Germany,** Mecklenburg-Vorpommern, Island of Rügen | 2006 | Winter | H5N1 | **Mute Swan, Whooper Swan**, Canada Goose | >350  (Globig et al. 2009) | Annual IWC winter counts summed to give a total value for the island and surrounding coastline.  (2000-2021) | **Frequency of counts:** Single annual count only  **Effects of bird movement:** Wintering populations, therefore potential for inter-annual variation due to immigration and emigration |
| **Germany,** Saxony-Anhalt, Helme Reservoir | 2007 | Breeding | H5N1 | **Black-necked Grebe** | >290  (Friedrich Loeffler Institute) | Annual counts at the reservoir during the breeding season  (2001-2020) | **Frequency of counts:** Single annual count only  **Effects of bird movement:**  Breeding population, fluctuation of numbers between years occurred in absence of HPAI, and may be due to other factors such as water levels. |
| **Germany,** Mecklenburg-Western Pomerania state | 2016 - 2017 | Breeding | H5N8 | **White-tailed Eagle** | >17  (Krone et al. 2018) | Annual nest counts for breeding pairs across the Mecklenburg-Western Pomerania state  (2000-2022) | **Frequency of counts:** Single annual count only  **Effects of bird movement:**  Count of breeding pairs across region, therefore expect limited impact of movements |
| **France,** Dombes region,  Ain department | 2006 | Winter | H5N1 | **Mute Swan** | >150  (WAHIS) | Annual IWC winter counts collected in two Ain communes, which were combined to produce a single total for the department  (1980-2022) | **Frequency of counts:** Single annual count only  **Effects of bird movement:**  Wintering populations, therefore potential for inter-annual variation due to immigration and emigration |
| **Slovenia,** Lendava gravel pits | 2017 | Winter | H5N8 | **Mute Swan** | >225  (WAHIS) | Annual January IWC counts summed across the 7 monitored gravel pits.  (2009 – 2022) | **Frequency of counts:** Single annual count only  **Effects of bird movement:**  Wintering populations, therefore potential for inter-annual variation due to immigration and emigration |
| **UK,** Dorset, Abbotsbury Swannery | 2005 – 2007,  2016 | Winter | H5N1 | **Mute Swan** | >100  (Recoveries recorded by Abbotsbury swannery) | Monthly WBS counts  (2000 – 2022) | **Frequency of counts:** Monthly counts  **Effects of bird movement:**  Wintering populations, therefore potential for inter-annual variation due to immigration and emigration |
| **Romania,** Constanta & Danube Delta | 2015 | Breeding | H5N1 | **Dalmatian Pelican** | >108  (Kiss et al. 2016) | Annual minimum and maximum counts of pairs were obtained from a minimum of two ultralight aircraft or drone surveys conducted during the breeding season, with the mean used as the annual estimate.  (2009 – 2024) | **Frequency of counts:** Two counts per breeding season  **Effects of bird movement:**  Breeding population with high site fidelity, therefore expect limited impact of movements |
| **Bulgaria,** Silistra Province, Srebarna Nature Reserve | 2015 | Breeding | H5N1 | **Dalmatian Pelican** | 27  (Ministry of agriculture data; WAHIS) | Annual census of breeding pairs via video surveillance or drone surveys.  (2010-2020) | **Frequency of counts:** Single annual count only  **Effects of bird movement:**  Breeding population with high site fidelity, therefore expect limited impact of movements |
| **Japan,** Kagoshima Prefecture, Izumi Crane Park | 2010 | Winter | H5N1 | **Hooded Crane, White-naped Crane** | >250  (Izumi Crane Park data) | Maximum count across the site, derived from between four to six single-day winter counts (Methods described in Lee, 2025)  (1950-2022) | **Frequency of counts:** Maximum counts used from multiple counts per winter (4-6)  **Effects of bird movement:**  Wintering populations, therefore potential for inter-annual variation due to immigration and emigration |
| **South Korea,** Jeonbuk province, Donglim Reservoir | 2014 | Winter | H5N8 | **Baikal Teal** | <200  (Young-Jun, pers. comm) | Annual winter count summed across the two locations (Donglim Reservoir and the Geum river in Jeonbuk Province), including only years when both sites were surveyed.  (2000-2022) | **Frequency of counts:** Single annual count only  **Effects of bird movement:**  Wintering populations, therefore potential for inter-annual variation due to immigration and emigration |
| **Algeria,** Ghardaia,  Sabkhat el Malah (Salt lake), El Menia province | 2016 | Winter | H7N1 | Ruddy Shelduck, Northern Shoveler, Marbled Teal, Black-winged Stilt, Common Coot, Common Moorhen,  Eurasian Teal, Ferruginous Pochard, Little Egret | >1,250  (WAHIS; National Network of Algerian Ornithological Observers) | IWC Winter counts, except January 2019, which represents the mean of two counts conducted during the 2018-2019 winter season.  (2006-2018) | **Frequency of counts:** Single annual count only  **Effects of bird movement:**  Wintering populations, therefore potential for inter-annual variation due to immigration and emigration |
| **Uganda,**  Lake Victoria | 2017 | Winter | H5N8 | **White-winged Tern** | >1,200  (Nantima et al. 2019) | Annual IWC counts in January-February  (2000-2023) | **Frequency of counts:** Single annual count only  **Effects of bird movement:**  Wintering populations, therefore potential for inter-annual variation due to immigration and emigration |
| **South Africa,** Eastern & western cape | 2016 - 2018 | Breeding | H5N8 | **Swift Tern, African Penguin, Cape Gannet, Cape Cormorant** | >7,500  (Roberts et al. 2023) | Annual breeding counts from survey locations along the coastline were summed to provide a total for the area.  (2009-2021) | **Frequency of counts:** Single annual count only  **Effects of bird movement:**  Breeding populations expected to have high site fidelity, therefore expect limited impact of movements |
| **Namibia,** Halifax Island | 2019 | Breeding | H5N8 | **African Penguin** | >350  (Molini et al. 2020) | Colony counts were derived from imagery of two (of three) colonies at mean intervals of 9-10 days. Counts were summed, and a negative binomial GLM was used to calculate the annual population index of a Penguin Year (1 July t- 20 June).  (08/2015 – 04/2023) | **Frequency of counts:** Regular repeated counts  **Effects of bird movement:**  Breeding populations expected to have high site fidelity, therefore expect limited impact of movements |
| **Iran,** Gilan Province, Bujaqh National Park | 2018 | Winter | H5N6 | **Gadwall**, Eurasian Wigeon, **Black-headed Gull**, **Pallas’s Gull** | >7,300  (Abbas Ashoori, pers. comm) | Annual IWC counts.  (2004-2023) | **Frequency of counts:** Single annual count only  **Effects of bird movement:**  Wintering populations, therefore potential for inter-annual variation due to immigration and emigration |
| **Iran,** Markazi province, Mighan wetland | 2017 | Winter | H5N8 | **Greylag Goose**, **Mallard**, **Common Crane**, **Common Coot**, Redshank, **Greater Flamingo**, Shelduck, Ruddy Shelduck | ~3000  (Hamid Amini, pers. comm) | Mid-January census counts  (2001-2022) | **Frequency of counts:** Single annual count only  **Effects of bird movement:**  Wintering populations, therefore potential for inter-annual variation due to immigration and emigration |

**Table S3- Virus and host-species traits extracted for each of the species-site cases** **that met the inclusion criteria**

| **Variable** | **Definition and levels** |
| --- | --- |
| 'Site’ | Local area or region in which the outbreak was reported.  *22 levels* |
| ‘Virus Clade’ | Virus clade for H5 viruses.  *4 levels: H5N1 2.2, H5N1 2.3.2.1, H5N8 2.3.4.4, H5NX 2.3.4.4b* |
| ‘Initial impact’ | Percentage change in population count following the initial outbreak.  *Continuous* |
| ‘Secondary HPAI’ | Whether further laboratory confirmed HPAI cases were detected in subsequent years within the post-outbreak period  *2 levels: No further detections, Further detections* |
| 'Group’ | A variable used to group species by phylogeny and ecology.  *6 levels: ‘waterfowl’ (Anseriformes), ‘flamingos & grebes’ (Mirandornithes), cranes & rails, hereafter ‘cranes’ (Gruiformes), ‘gulls & terns’ (Laridae), penguins, Suliformes, and pelicans, hereafter ‘seabirds & pelicans’ (Aequornithes), ‘raptors’* |

**Table S4- Species groupings for analysis and description.**

| **Waterfowl** | **Raptors** | **Gulls & terns** | **Cranes** | **Flamingo & grebes** | **Seabirds** |
| --- | --- | --- | --- | --- | --- |
| Baikal teal | Peregrine Falcon | Black-headed gull | Common Crane | Black-necked Grebe | Cape Gannet |
| Common Pochard | White-tailed Eagle | Great Black-backed Gull | White-naped Crane | Greater Flamingo | Dalmatian Pelican |
| Eurasian Wigeon |  | Pallas’s Gull | Hooded Crane |  | African Penguin |
| Gadwall |  | Swift Tern | Common Coot |  | Cape Cormorant |
| Greylag Goose |  | White-winged Tern |  |  |  |
| Mallard |  |  |  |  |  |
| Mute Swan |  |  |  |  |  |
| Tufted Duck |  |  |  |  |  |
| Whooper Swan |  |  |  |  |  |

**Fig S1- Visual summary of three measures of outbreak impact extracted.** Blue lines represent the series of population counts over time. In plot A, red points indicate the counts pre- and post-outbreak that are used to derive the initial population change. In plot B, red dotted lines represent the average population counts pre- and post-outbreak. In plot C, the red dotted line represents the trend if unchanged from prior to the outbreak. The red arrow represents the difference calculated between the pre-outbreak trend and the post-outbreak trend.
