# Supplementary material for: Characterising the longer-term impacts of highly pathogenic avian influenza outbreaks prior to 2020 on wild bird populations
Source: PLoS One. 2026 Nov 11;21(11):e0360235. doi: 10.1371/journal.pone.0360235 (PMC13754095; doi:10.1371/journal.pone.0360235)
Supplement: Supplementary file 2 [file pone.0360235.s002.docx]

**Supporting Information**

**Characterising the longer-term impacts of highly pathogenic avian influenza outbreaks prior to 2020 on wild bird populations**

**S2 Text: Results**

**S2.1. Initial outbreak impacts**

The distribution of carcass counts across all species-site cases and observed mortality (carcass counts/ population count) are shown in Fig S2A and B. For cases where initial declines in population count occurred, no significant association was found between observed mortality, and initial population count change (Fig S2C).

**Fig S2- Comparison of observed mortality and population count change.** Distribution of A) carcass counts and B) observed % mortality (Carcass count/baseline population count). C) No correlation was detectable between observed % mortality and initial population count change, for cases where population counts decreased. The vertical dotted line in panel B indicates 100% mortality- for cases above this reported mortality was higher than the baseline population count.

**S2.2. Summary of model outputs for each species-site case**

**Table S5- Summary of outbreak impact measures across all 40 species-cases.** Results in orange show reductions (in initial abundance, long-term population count or population growth rate). Results in bold are significant at the p<0.05 level.

| **Species** | **Location** | **Initial change in population count (%)** | **Change in long term population count (%)** | **P-value for population count change** | **Change in trend (%)** | **P-value for trend change** | **Growth rate post outbreak** (Values <1 population decline, >1 increase) |
| --- | --- | --- | --- | --- | --- | --- | --- |
| **African Penguin** | Halifax island | -46.96 | **-42.6** | <0.001 | -13.1 | 0.1174 | 0.998 |
| **African Penguin** | Western and Eastern Cape provinces | -17.64 | **-39.72** | <0.001 | -6.98 | 0.056 | 0.905 |
| **Baikal Teal** | South West Republic of Korea | 1140 | 16.54 | 0.6728 | **30.34** | 0.0318 | 0.97 |
| **Black-headed Gull** | Bujaqh National Park | -96.21 | 300.77 | 0.1699 | 55.16 | 0.4311 | 1.559 |
| **Black-necked Grebe** | Helme reservoir | -16.09 | 61.85 | 0.3513 | **-122.68** | 0.0158 | 0.979 |
| **Cape Cormorant** | Western and Eastern Cape provinces | -25.53 | -19.61 | 0.356 | -10.79 | 0.5487 | 0.977 |
| **Cape Gannet** | Western and Eastern Cape provinces | -12.83 | 0.89 | 0.9014 | 9.68 | 0.076 | 1.095 |
| **Common Coot** | Mighan wetlands | 9900 | -69.11 | 0.3127 | **73.67** | 0.0429 | 1.149 |
| **Common Crane** | Mighan wetlands | -33.88 | **-48.28** | 0.0063 | 1.5 | 0.911 | 0.972 |
| **Common Pochard** | Lake Constance | -60.25 | **-37.04** | 0.0237 | -13.69 | 0.1101 | 1.051 |
| **Common Pochard** | Lake Constance | 33.6 | -26.7 | 0.0512 | -5.21 | 0.2812 | 0.874 |
| **Dalmatian Pelican** | Danube delta | -38.79 | 13.79 | 0.4169 | 7.5 | 0.4453 | 1.088 |
| **Dalmatian Pelican** | Srebarna lake | 18.64 | -29.3 | 0.1476 | 5.67 | 0.4536 | 0.866 |
| **Eurasian Wigeon** | Friesland | 37.84 | 21.59 | 0.1247 | 3.98 | 0.6797 | 1.079 |
| **Eurasian Wigeon** | Noord-Holland | 14.85 | 10.99 | 0.2673 | -10.08 | 0.0925 | 0.961 |
| **Gadwall** | Bujaqh National Park | -87.79 | -35.67 | 0.2628 | **71.0** | 0.0325 | 1.766 |
| **Great Black-backed Gull** | N2000 Wadden Sea | -40.14 | -24.46 | 0.2092 | -17.33 | 0.3341 | 0.868 |
| **Greater Flamingo** | Mighan wetlands | -33.33 | -79.85 | 0.2451 | 121.93 | 0.1687 | 1.647 |
| **Greylag Goose** | Mighan wetlands | 186.37 | **252.12** | 0.0053 | -5.58 | 0.8073 | 0.976 |
| **Hooded Crane** | Izumi Crane Park | -4.15 | **20.7** | 0.0105 | 2.54 | 0.2798 | 1.052 |
| **Mallard** | Lake Constance | -12.68 | -2.47 | 0.7847 | -6.97 | 0.1587 | 1.004 |
| **Mallard** | Mighan wetlands | 822.08 | -47.47 | 0.3551 | 28.23 | 0.1537 | 0.808 |
| **Mute Swan** | Abbotsbury Swannery | -2.88 | **17.51** | 0.0416 | 0.28 | 0.9456 | 0.983 |
| **Mute Swan** | Dombes region | -22.05 | 24.64 | 0.1824 | -15.52 | 0.0546 | 0.965 |
| **Mute Swan** | Lake Constance | -15.6 | **17.51** | 0.0405 | 4.55 | 0.0842 | 1.065 |
| **Mute Swan** | Lake Constance | 4.68 | **33.98** | <0.001 | **2.87** | 0.0279 | 1.061 |
| **Mute Swan** | Lendava gravel pits | 86.78 | -22.63 | 0.5367 | -32.73 | 0.2975 | 0.846 |
| **Mute Swan** | Reugen island | -34.53 | 55.08 | 0.1249 | -19.69 | 0.2524 | 0.971 |
| **Pallas's Gull** | Bujaqh National Park | -100 | 11.06 | 0.8068 | 19.88 | 0.5632 | 1.246 |
| **Peregrine Falcon** | N2000 Wadden Sea | -28.4 | **-21.07** | 0.0211 | -1.44 | 0.8521 | 1.01 |
| **Swift Tern** | Western and Eastern Cape provinces | 2.28 | -43.91 | 0.0573 | 21.02 | 0.4503 | 1.193 |
| **Tufted Duck** | Lake Constance | -36.75 | **-28.64** | 0.0035 | 4.24 | 0.3981 | 1.056 |
| **Tufted Duck** | Lake Constance | -9.52 | **-42.16** | 0.0016 | -1.49 | 0.8245 | 0.931 |
| **Tufted Duck** | Großer Plöner See | -52.94 | -25.56 | 0.2577 | **-36.83** | <0.001 | 0.919 |
| **Tufted Duck** | N2000 Markermeer & Ijmeer | -21.21 | **-45.03** | 0.0067 | -3.28 | 0.8212 | 0.927 |
| **Tufted Duck** | N2000 Veluwerandmeren | -28.38 | -13.91 | 0.4066 | -15.08 | 0.2735 | 0.947 |
| **White-naped Crane** | Izumi Crane Park | 1.78 | **35.68** | 0.022 | -1.68 | 0.7946 | 0.984 |
| **White-tailed Eagle** | Mecklenburg-Western Pomerania state | 7.55 | **29.29** | <0.001 | -1.57 | 0.2553 | 1.037 |
| **White-winged Tern** | Lake Victoria | -98.73 | -70.88 | 0.2168 | 33.13 | 0.7059 | 1.521 |
| **Whooper Swan** | Reugen island | -27.31 | 54.96 | 0.1425 | -20.36 | 0.2422 | 0.948 |

**S2.3. Sensitivity analysis of model outputs to number of years used for estimates**

In the main text, long-term impacts are presented across population counts using a window of up to 7 years post-outbreak, relative to 7 years pre-outbreak. Fig S4 shows the sensitivity of the model outputs to the time period used post-outbreak. All models were run using between 3 and 10 counts post-outbreak to estimate post-outbreak abundance and trend. For each species-site case, model outputs are plotted against the number of years. Any cases where points (model outputs) are on either side of the black dotted line, indicates a change in model estimate from a positive to negative value depending on the number of years used.

In most cases, the direction of change for abundance (increase or decrease post-outbreak) was not sensitive to the number of years used to estimate the post-outbreak trend. However, in two species-site cases (in Cape gannet and Dalmatian pelican), if only 3 years of points were used, model results indicated a reduction in post-outbreak abundance, whereas using 4 or more years, abundances increased post-outbreak. For other cases, while the direction of abundance change remained constant, the magnitude of change was sensitive to number of years used e.g. in Black headed gull.

**Fig S3- Sensitivity of predicted change in longer-term population count to the number of years used to estimate post-outbreak, relative to constant pre-outbreak baseline (7 years).** The black dotted line indicates no change in population count, the lower red dotted line indicates population extinction and the higher red dotted line indicates a doubling of the population count. Therefore, where points are consistently below the black line, this indicates population reduction, whereas values above this indicates population increase. Red points indicate a decrease in predicted population abundance post-outbreak, and blue points an increase.

For predicted trend, the direction of the result was more sensitive to the number of years used, with 11 of the 40 outbreaks showing a change in direction depending on the number of years used.

**Fig S4- Sensitivity of predicted change in trend to the number of years used for estimate post-outbreak relative to constant pre-outbreak baseline (7 years).** The black dotted line indicates no change in trend. Therefore, where points are consistently below the black line, this indicates a decrease in population trend post-outbreak, whereas above this indicates an increase in trend.

**S2.4 Statistical models of predictors of outbreak impact**

*Initial impacts*

**Fig S5- Residual plots to evaluate model assumptions for mixed effects model of initial impact on population count.**

*Long-term population count change*

**Fig S6- Residual plots to evaluate model assumptions for linear model of longer-term population count change**

**Fig S7- Effect sizes from linear model for longer-term population count change**

*Long-term trend change*

**Fig S8- Residual plots to evaluate model assumptions for linear model of long-term trend**

**Fig S9- Effect sizes from linear model for trend change**

**References**

1. Olsen, B., Munster, V. J., Wallensten, A., Waldenström, J., Osterhaus, A. D., & Fouchier, R. A. (2006). Global patterns of influenza A virus in wild birds. *science*, *312*(5772), 384-388.
2. Gauthier‐Clerc, M., Lebarbenchon, C., & Thomas, F. (2007). Recent expansion of highly pathogenic avian influenza H5N1: a critical review. *Ibis*, *149*(2), 202-214.
3. Kleyheeg, E., Slaterus, R., Bodewes, R., Rijks, J. M., Spierenburg, M. A., Beerens, N., ... & van der Jeugd, H. P. (2017). Deaths among wild birds during highly pathogenic avian influenza A (H5N8) virus outbreak, the Netherlands. *Emerging infectious diseases*, *23*(12), 2050.
4. Lycett, S. J., Duchatel, F., & Digard, P. (2019). A brief history of bird flu. *Philosophical Transactions of the Royal Society B*, *374*(1775), 20180257.
5. Krone, O., Globig, A., Ulrich, R., Harder, T., Schinköthe, J., Herrmann, C., ... & Beer, M. (2018). White-tailed sea eagle (Haliaeetus albicilla) die-off due to infection with highly pathogenic avian influenza virus, subtype H5N8, in Germany. *Viruses*, *10*(9), 478.
6. Globig, A., Staubach, C., Beer, M., Köppen, U., Fiedler, W., Nieburg, M., ... & Harder, T. C. (2009). Epidemiological and ornithological aspects of outbreaks of highly pathogenic avian influenza virus H5N1 of Asian lineage in wild birds in Germany, 2006 and 2007. *Transboundary and emerging diseases*, *56*(3), 57-72.
7. Kiss, B. J., Alexe, V., Dorosencu, A. C., Ceico, T., Kiss, N. B., & Marinov, M. E. (2016). Recent data on the Danube Delta (Romania) avifauna from the 2014 and 2015 summer seasons. *Acrocephalus*, *37*(168-169), 85.
8. Nantima, N., Ademun, A. R. O., Sentumbwe, J., Ilukor, J., Kirumira, M. M., Muwanga, E., ... & Von Dobschuetz, S. (2019). Managing the challenges of a highly pathogenic avian influenza H5N8 outbreak in Uganda: a case study. *Revue Scientifique et Technique (International Office of Epizootics)*, *38*(1), 225-237.
9. Roberts, L. C., Abolnik, C., Waller, L. J., Shaw, K., Ludynia, K., Roberts, D. G., ... & Abernethy, D. (2023). Descriptive epidemiology of and response to the high pathogenicity avian influenza (H5N8) epidemic in South African coastal seabirds, 2018. *Transboundary and Emerging Diseases*, *2023*(1), 2708458.
10. Molini, U., Aikukutu, G., Roux, J. P., Kemper, J., Ntahonshikira, C., Marruchella, G., ... & Dundon, W. G. (2020). Avian influenza H5N8 outbreak in African penguins (Spheniscus demersus), Namibia, 2019. *The Journal of Wildlife Diseases*, *56*(1), 214-218.
11. Lee, H. (2025). Updated global population estimates of three endemic crane species in Northeast Asia: wintering status and conservation implications in Korea and Japan. *Diversity*, *17*(10), 682.
